# Supplementary figures and images for: Simian Immunodeficiency Virus-Based Virus-like Particles Are an Efficient Tool to Induce Persistent Anti-SARS-CoV-2 Spike Neutralizing Antibodies and Specific T Cells in Mice
Source: Vaccines (Basel). 2025 Feb 21;13(3):216. doi: 10.3390/vaccines13030216 (PMC11945333; doi:10.3390/vaccines13030216)

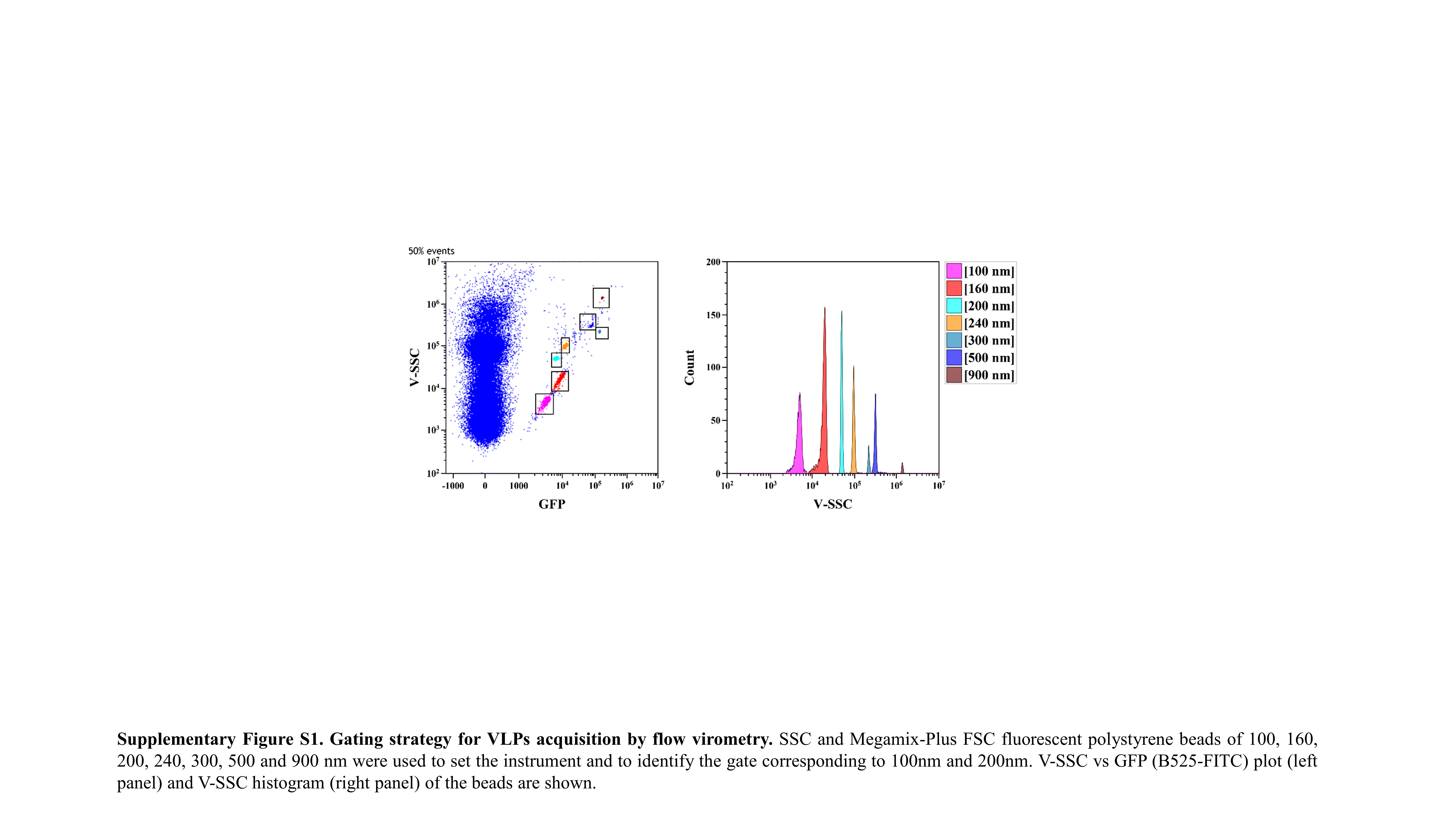

Supplement: Supplementary file 1 [file vaccines-13-00216-s001.zip › Supplementary Figure S1.jpg]

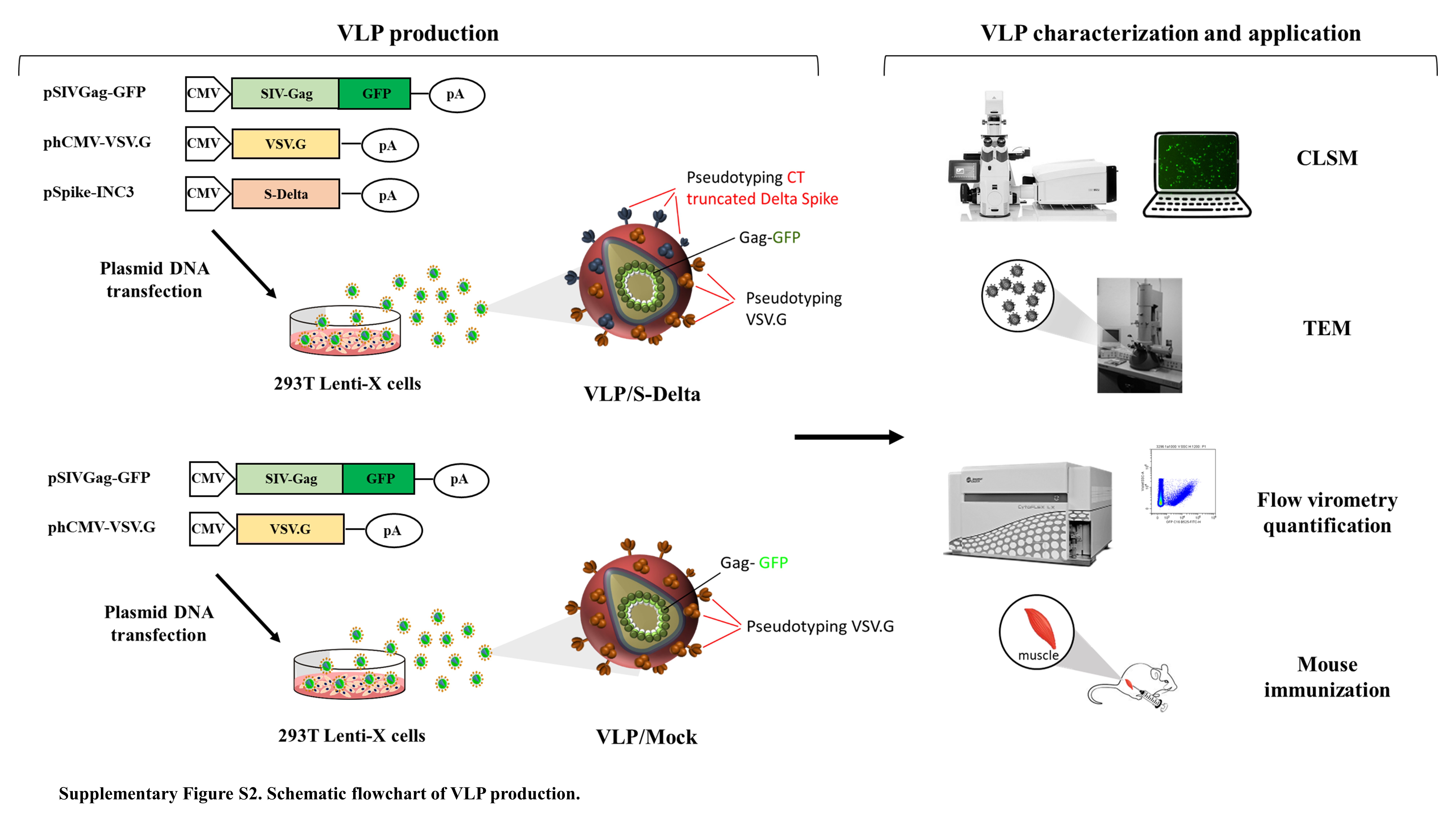

Supplement: Supplementary file 1 [file vaccines-13-00216-s001.zip › Supplementary Figure S2.jpg]
